# A new perspective on ATR's role in translesion synthesis

**DOI:** 10.1101/gad.353773.126

**Published:** 2026-07-01

**Authors:** Laura A. Lindsey-Boltz, Aziz Sancar

**Affiliations:** Department of Biochemistry and Biophysics, University of North Carolina School of Medicine, Chapel Hill, North Carolina 27599, USA

**Keywords:** DNA repair, ATR, fork stalling, UV damage, translesion synthesis

## Abstract

During DNA replication, the physical and functional coupling of DNA unwinding by the CMG helicase with the DNA synthesis components of the replisome promotes highly efficient replication of the genome. What happens when the replication fork stalls at a DNA lesion? Do translesion synthesis (TLS) DNA polymerases operate in close coordination with the replisome or function independent of it; e.g., in daughter strand gaps? Using noncancer human cell lines, Yoon and colleagues (doi:10.1101/gad.353474.125) show that TLS operates in close coordination with the replisome and that ATR stabilizes the replisome at stalled replication forks; thus, TLS protects against chromosomal instability.

## Abstract (precis)

In this Outlook, Lindsey-Boltz and Sancar discuss a study in this issue of *Genes & Development* by Yoon et al. that reports that translesion synthesis is coupled with the replisome to achieve error-free DNA replication through stalled replication forks at DNA lesions. The mechanisms by which translesion synthesis proceeds depend on ATR kinase and involve different DNA polymerase complexes that enable lagging strand DNA replication while preserving chromosomal stability.

Mammalian TLS DNA polymerases (Pols), including Polη, Polθ, and Rev1-Polζ, are highly specialized for replicating through a particular type of DNA lesion. However, despite their high specificity for the DNA lesion, purified TLS Pols synthesize DNA opposite DNA lesions with a high error rate. The high error proneness of purified TLS Pols has led to the widely held assumption that cellular TLS Pols contribute to somatic mutations and hence to cancer formation. Studies from the Prakash group (Yoon et al. 2019, 2025, 2026) have made significant advances in dismantling this view.

Although purified TLS Pols exhibit high error rates in DNA synthesis, TLS in human cells operates in a predominantly error-free manner that is achieved in part by the incorporation of the TLS Pol into a multiprotein ensemble that raises the fidelity of the TLS Pol (Yoon et al. 2025). Polη-dependent TLS opposite UV-induced cyclobutane pyrimidine dimers (CPDs) is error-free in human cells, and Polη deficiency confers increased UV-induced mutations and skin cancer (Yoon et al. 2025). However, in contrast to error-free bypass of CPDs by Polη, Polθ promotes error-prone TLS opposite CPDs and also opposite the much less prevalent UV lesion (6-4) pyrimidine-pyrimidone photoproduct; hence, the UV-induced mutations in skin cells derive from Polθ’s role in TLS (Yoon et al. 2019). Thus, one might predict that error-prone TLS by Polθ would promote sunlight-induced skin carcinogenesis. Surprisingly, however, UV-induced skin cancer incidence is also elevated in Polθ-deficient mice (Yoon et al. 2019). The study helped to resolve this apparent paradox by demonstrating that both error-free TLS mediated by Polη and error-prone TLS mediated by Polθ protect against tumorigenesis, not by minimizing mutagenesis per se, but by preventing replication fork collapse and the resulting chromosomal instability.

The elucidation of how TLS promotes efficient replication through DNA lesions, prevents fork collapse, and protects against chromosomal instability requires an understanding of the mechanisms by which TLS is coordinated when the replisome encounters a DNA lesion. Although studies in yeast (Daigaku et al. 2010; Karras and Jentsch 2010; Wong et al. 2020) and in human cancer cells (Taglialatela et al. 2021; Tirman et al. 2021) have implicated a postreplicative gap-filling model of TLS, in the study in this issue of *Genes & Development*, Yoon et al. (2026) present strong evidence that TLS in noncancer human cells operates in concert with the replisome and that ATR kinase stabilizes the replisome at the stalled fork. To demonstrate the coordination of TLS with the replisome, the investigators show that both the replisome and TLS components are found in UV-induced foci as well as in isolated chromatin fractions from UV-irradiated human fibroblasts. Additionally, they provide direct evidence for elevated levels of replisome and TLS components at replication forks (RFs) stalled at UV lesions compared to actively replicating unirradiated cells.

When ATR kinase is inhibited, Yoon et al. (2026) found that the CMG and DNA synthesis components of the replisome disassemble from forks stalled at UV lesions and that RF progression through UV lesions in ATR-deficient cells is reduced similar to that which occurs in the absence of TLS in Polη- and Polθ-deficient cells (Fig. 1). This provides strong evidence for the pivotal role of ATR in maintaining the replisome at the stalled fork and in coordinating TLS with the replisome. The large reduction in RF progression in ATR-deficient cells further suggests that a DNA lesion on either the leading or lagging strand leads to stalling of the replication fork and to stabilization of the replisome by ATR, suggesting that TLS on both DNA strands is coordinated with the replisome by ATR.

**Figure 1.**
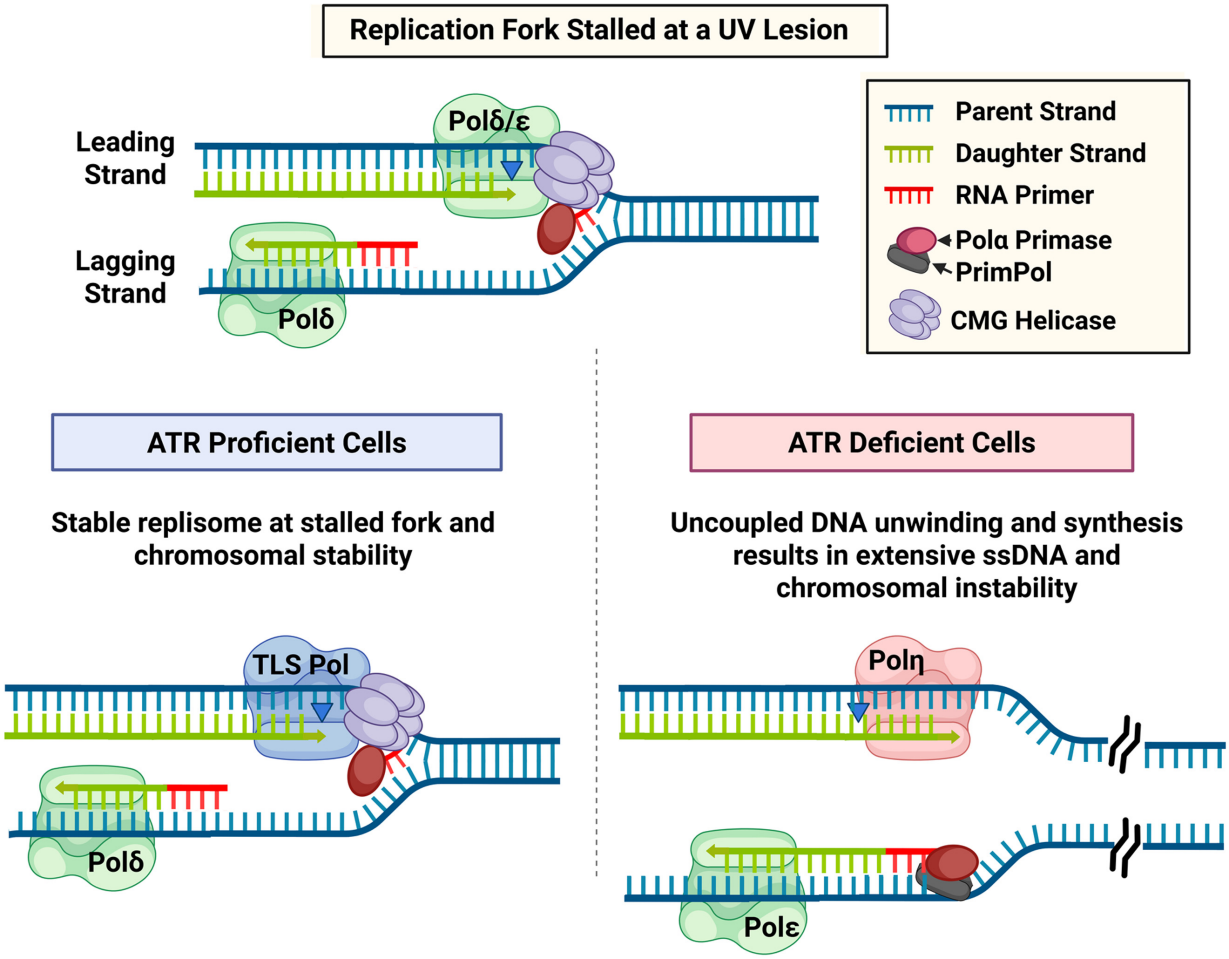
A model for replication of leading and lagging DNA strands at a replication fork stalled at a UV lesion in ATR-proficient and ATR-deficient cells. During normal DNA replication, the CMG helicase unwinds duplex DNA to provide a ssDNA substrate for DNA synthesis by the replicative Pols (δ and ε). When DNA replication becomes stalled at a UV lesion (blue triangle), ATR kinase activation stabilizes the replisome in many ways, including facilitating the swap of Polδ/ε for a TLS pol. In the absence of ATR-dependent phosphorylation of CMG and the DNA synthesis components of the replisome, the CMG helicase becomes uncoupled from DNA synthesis, and there is unrestricted unwinding of the duplex. Consequently, TLS and replication mechanisms are altered drastically in ATR-deficient cells, and one of the most surprising findings of the study by Yoon et al. (2026) is that lagging strand synthesis at forks stalled at UV lesions in ATR-deficient cells is carried out by the action of the newly identified PrimPol–PolA1/PolA2 Polα complex in primer synthesis and extension of synthesis by Polε rather than by the combined actions of PolA1/PolA2/PRIM1/PRIM2 Polα in primer synthesis and Polδ in extension of synthesis, as occurs in ATR-proficient cells.

Because Polη-mediated TLS occurs in ATR-deficient cells, this allowed Yoon et al. (2026) to demarcate the means that ATR-deficient cells utilize to replicate through UV lesions. Interestingly, the mechanism of lagging strand synthesis in UV-damaged ATR-inhibited cells is altered in unexpected ways compared with those in ATR-proficient cells. For example, lagging strand synthesis at forks stalled at UV lesions in ATR-deficient cells is carried out by the action of the newly identified PrimPol–PolA1/PolA2 Polα complex in primer synthesis and extension of synthesis by Polε rather than by the combined actions of PolA1/PolA2/PRIM1/PRIM2 Polα in primer synthesis and Polδ in extension of synthesis, as occurs in ATR-proficient cells. These surprising results identify an entirely new means of lagging strand DNA replication.

Finally, the study by Yoon et al. (2026) demonstrates the relevance of coupling of TLS with the replisome for genome stability. Although in ATR-deficient cells Polη-mediated TLS operates uncoupled from the replisome, unrestrained fork unwinding by the CMG helicase results in increased single-stranded DNA in UV-irradiated ATR-deficient cells and an increase in chromosomal instability. Thus, the adaptation of TLS Pols to perform predominantly error-free replication through DNA lesions and the coupling of TLS with the replisome to avoid chromosomal instability raise the possibility that replisome-associated TLS Pol-dependent mechanisms promote replication advancement not only through DNA lesions but also through the various other types of chromosomal impediments. Therefore, such replisome-associated mechanisms would make a prominent contribution to the completion of genome replication and to genome stability. It is also of interest to note that while TLS mechanisms in human cells have been adapted to protect against genome instability by raising the fidelity of TLS Pols and by coupling TLS with the replisome, TLS mechanisms in cancer cells, which operate in postreplicational gaps and depend on PrimPol and Rev1-Polζ irrespective of the DNA lesion, would contribute to genome instability and aid in metastatic progression.

## References

[GAD353773LINC1] Daigaku Y, Davies AA, Ulrich HD. 2010. Ubiquitin-dependent DNA damage bypass is separable from genome replication. Nature 465: 951–955. 10.1038/nature09097 20453836 PMC2888004

[GAD353773LINC2] Karras GI, Jentsch S. 2010. The *RAD6* DNA damage tolerance pathway operates uncoupled from the replication fork and is functional beyond S phase. Cell 141: 255–267. 10.1016/j.cell.2010.02.028 20403322

[GAD353773LINC3] Taglialatela A, Leuzzi G, Sannino V, Cuella-Martin R, Huang JW, Wu-Baer F, Baer R, Costanzo V, Ciccia A. 2021. REV1-Polzeta maintains the viability of homologous recombination-deficient cancer cells through mutagenic repair of PRIMPOL-dependent ssDNA gaps. Mol Cell 81: 4008–4025.e7. 10.1016/j.molcel.2021.08.016 34508659 PMC8500949

[GAD353773LINC4] Tirman S, Quinet A, Wood M, Meroni A, Cybulla E, Jackson J, Pegoraro S, Simoneau A, Zou L, Vindigni A. 2021. Temporally distinct post-replicative repair mechanisms fill PRIMPOL-dependent ssDNA gaps in human cells. Mol Cell 81: 4026–4040.e8. 10.1016/j.molcel.2021.09.013 34624216 PMC8555837

[GAD353773LINC5] Wong RP, García-Rodríguez N, Zilio N, Hanulova M, Ulrich HD. 2020. Processing of DNA polymerase-blocking lesions during genome replication is spatially and temporally segregated from replication forks. Mol Cell 77: 3–16.e4. 10.1016/j.molcel.2019.09.015 31607544

[GAD353773LINC6] Yoon J-H, McArthur MJ, Park J, Basu D, Wakamiya M, Prakash L, Prakash S. 2019. Error-prone replication through UV lesions by DNA polymerase theta protects against skin cancers. Cell 176: 1295–1309.e15. 10.1016/j.cell.2019.01.023 30773314 PMC6453116

[GAD353773LINC7] Yoon J-H, Sellamuthu K, Prakash L, Prakash S. 2025. WRN and WRNIP1 ATPases impose high fidelity on translesion synthesis by Y-family DNA polymerases. eLife 14: RP106934. 10.7554/eLife.106934 40900148 PMC12408069

[GAD353773LINC8] Yoon J-H, Sellamuthu K, Johnson RE, Prakash L, Prakash S. 2026. Coupling of translesion synthesis with the replisome stabilized at stalled replication forks by ATR. Genes Dev (this issue). 10.1101/gad.353474.125 42140672

